# Supplementary material for: Entangled spin-polarized excitons from singlet fission in a rigid dimer
Source: Nat Commun. 2023 Mar 2;14:1180. doi: 10.1038/s41467-023-36529-6 (PMC9977721; doi:10.1038/s41467-023-36529-6)
Supplement: Supplementary file 1 — Supplementary Information [file 41467_2023_36529_MOESM1_ESM.pdf]

Supplementary Information

Entangled Spin-polarized Excitons from Singlet Fission in a Rigid Dimer

Ryan D. Dill[1,†], Kori E. Smyser[1,†], Brandon K. Rugg[2], Niels H. Damrauer[1,3,*], and Joel D. Eaves[1,3,*]

[1]Department of Chemistry, University of Colorado Boulder, Boulder, CO, 80309, USA.

[2]National Renewable Energy Laboratory, 15013 Denver West Parkway, Golden, CO, 80401, USA.

[3]Renewable and Sustainable Energy Institute, University of Colorado Boulder, Boulder, CO, 80309, USA.

[†]These authors contributed equally to this work.

[*]Corresponding author(s). E-mail(s): niels.damrauer@colorado.edu; joel.eaves@colorado.edu

Contents

1. Materials and Methods **S2**
- 1.1. Materials . . . S2
- 1.2. Transient Absorption Spectroscopy . . . S2
- 1.3. trEPR Spectroscopy . . . S6

2. Supplementary Text **S8**
- 2.1. The intersystem crossing triplet trEPR powder spectrum for TIPS-Pc . . . S8
- 2.2. Rabi oscillations and the Hankel transform . . . S8
  - 2.2.1. Hankel Transform . . . S10
  - 2.2.2. Spectrum Estimation . . . S11
- 2.3. The Q0 model for the initial population . . . S14
- 2.4. The spin hamiltonian . . . S14
- 2.5. The inter-chromophore anisotropic interaction . . . S15
  - 2.5.1. Deriving the *JDE* model with non-zero *X* . . . S15
- 2.6. Calculating EPR spectra . . . S16
  - 2.6.1. Choice of basis for the triplet pair . . . S17
- 2.7. Best-fit parameters by simulated annealing . . . S18

Supplementary Note 1. Materials and Methods

1.1 Materials

TIPS-BP1′ was prepared and purified as described previously[1]. Spectroscopic samples were prepared in 2-methyltetrahydrofuran (≥ 99%, anhydrous, inhibitor free, Sigma-Aldrich), which was stored in a glovebox under nitrogen atmosphere and used as received. TIPS-Pentacene (TIPS-Pc) was purchased from Sigma-Aldrich and used as received.

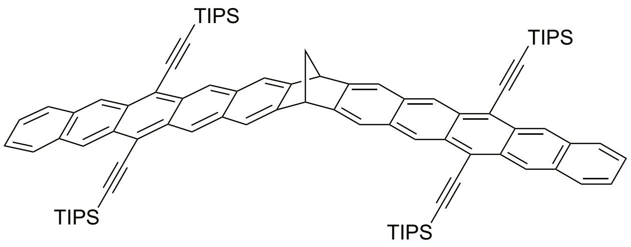

Supplementary Fig. 1: The structure of TIPS-BP1′. TIPS = triisopropylsilyl (Si(i-Pr)$_3$).

1.2 Transient Absorption Spectroscopy

Transient absorption (TA) spectroscopy of TIPS-BP1′ in room temperature toluene has been previously discussed[2]. We have also collected TA data in mTHF at 102 K, and these demonstrate similar dynamics (Supplementary Fig. 2). TA data were collected on a commercially available spectrometer (Ultrafast Systems, EOS) equipped with a continuous flow cryostat (Janis, STVP-100). The sample was prepared under a nitrogen atmosphere in a glovebox and flame sealed under vacuum in an ampoule made from a glass test tube. Excitation was centered at 640 nm, with pump fluence < 1 mJ/cm^2. The data show the well-known ESA peaked at 519 nm, as well as its vibronic progression, which we have previously assigned to the ^{1}TT state and form on a picosecond timescale. Ground state bleach features centered at 590 nm and 642 nm are also prominent. The spectral evolution can be modeled with a single exponential decay to baseline with a 145 ± 1 ns time constant (uncertainty range is a 95% confidence interval computed using MATLAB's nlparci function). This decay to baseline of the EPR silent ^{1}TT highlights that the observed trEPR signals discussed in the manuscript are from small populations.

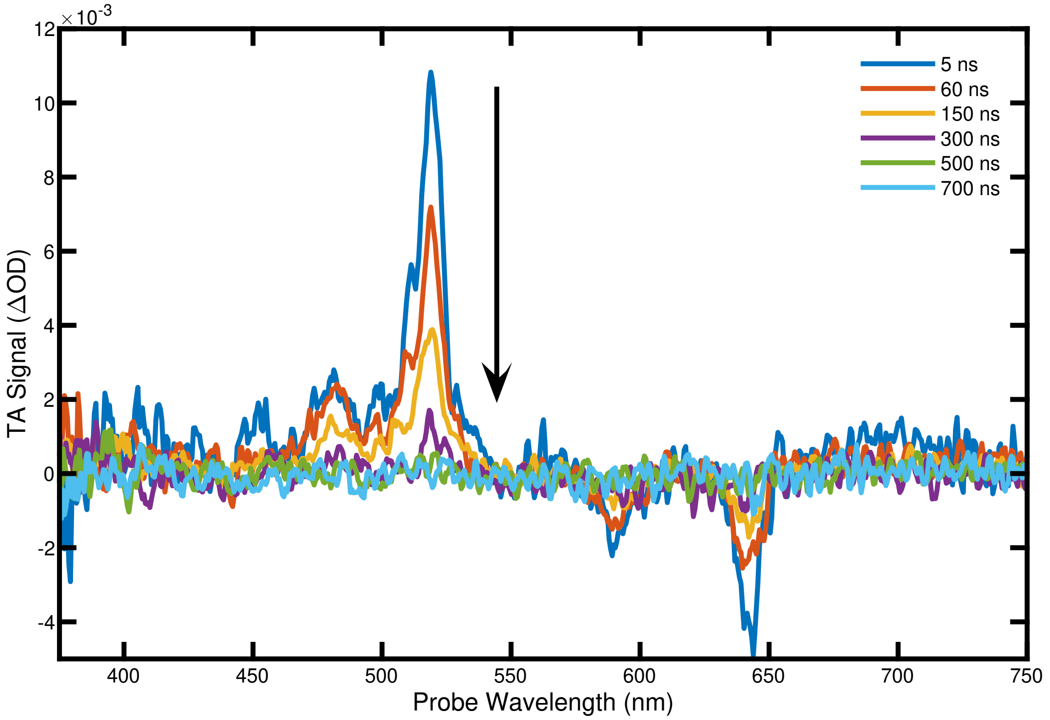

Supplementary Fig. 2: Nanosecond TA Spectra of TIPS-BP1′ in mTHF at 102 K. ^{1}TT forms within a few picoseconds[2]. On a nanosecond timescale, the TA dynamics mainly show decay of ^{1}TT to the ground state (black arrow). Under these conditions, the long-lived states probed in the trEPR experiment are not seen above the noise, suggesting the observed trEPR signals are from small populations in those states. As described in the text, the spectral evolution can be modeled with a single exponential decay with a time constant of 145 ns. Using single-wavelength measurements at the peak of the 519 nm excited state absorption (ESA), we observe temperature independence in the decay within the temperature regime of ≈110K to 77K (see Supplementary Figs. 4 and 3).

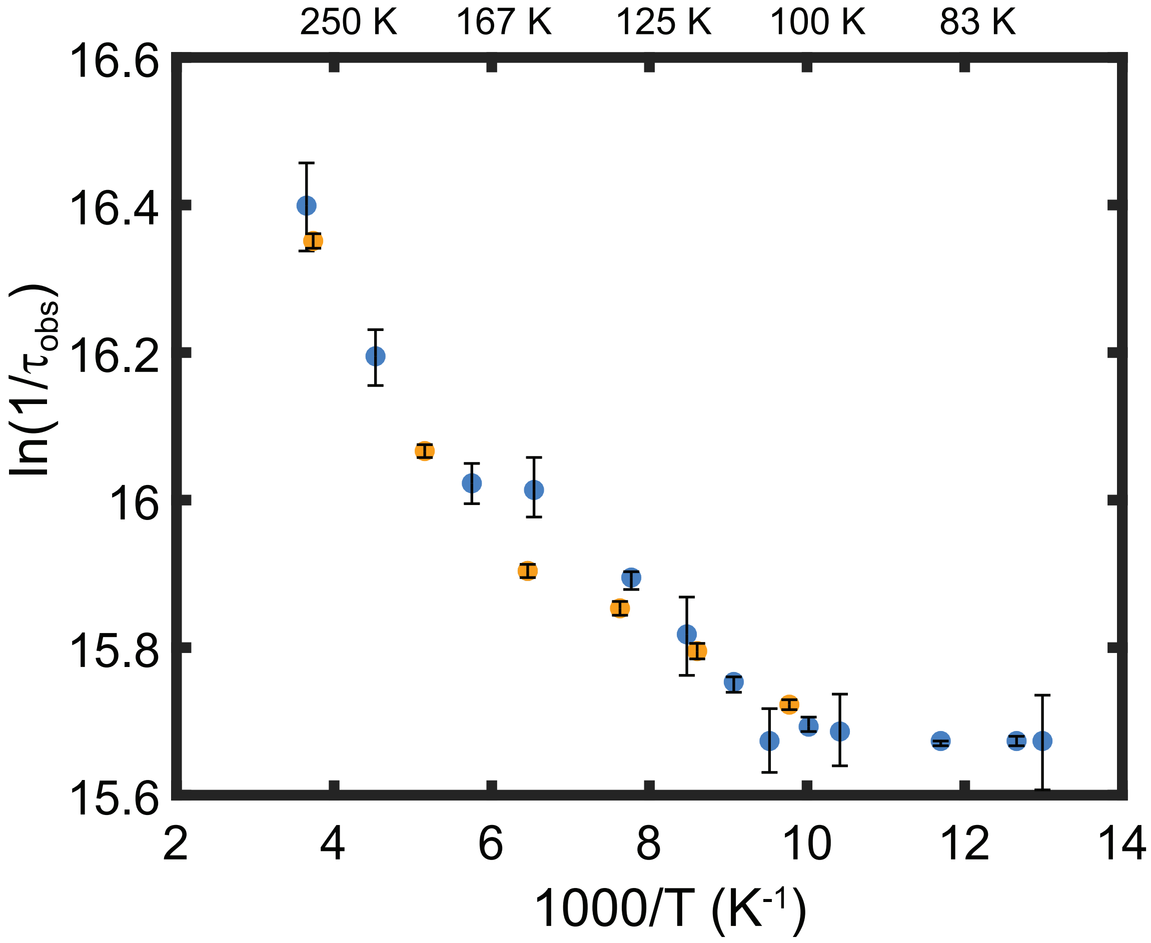

Supplementary Fig. 3: Arrhenius plot of [1]TT decay for TIPS-BP1′ in mTHF. Orange data points are from global fits of wavelength- and time-resolved data like that presented in Supplementary Fig. 2. Blue data points are from fits of single wavelength data sets collected on a separate TA setup. Error bars are 95% confidence interval computed from the fit Jacobian using MATLAB's nlparci function. At low temperatures (below ≈ 130 K), the spectral evolution can be modeled with a single exponential decay whose time constant is approximately temperature independent within the temperature range of ≈ 110K to 77K, as seen by the plateau at high values of 1/T (example kinetics are shown in Supplementary Fig. 4)

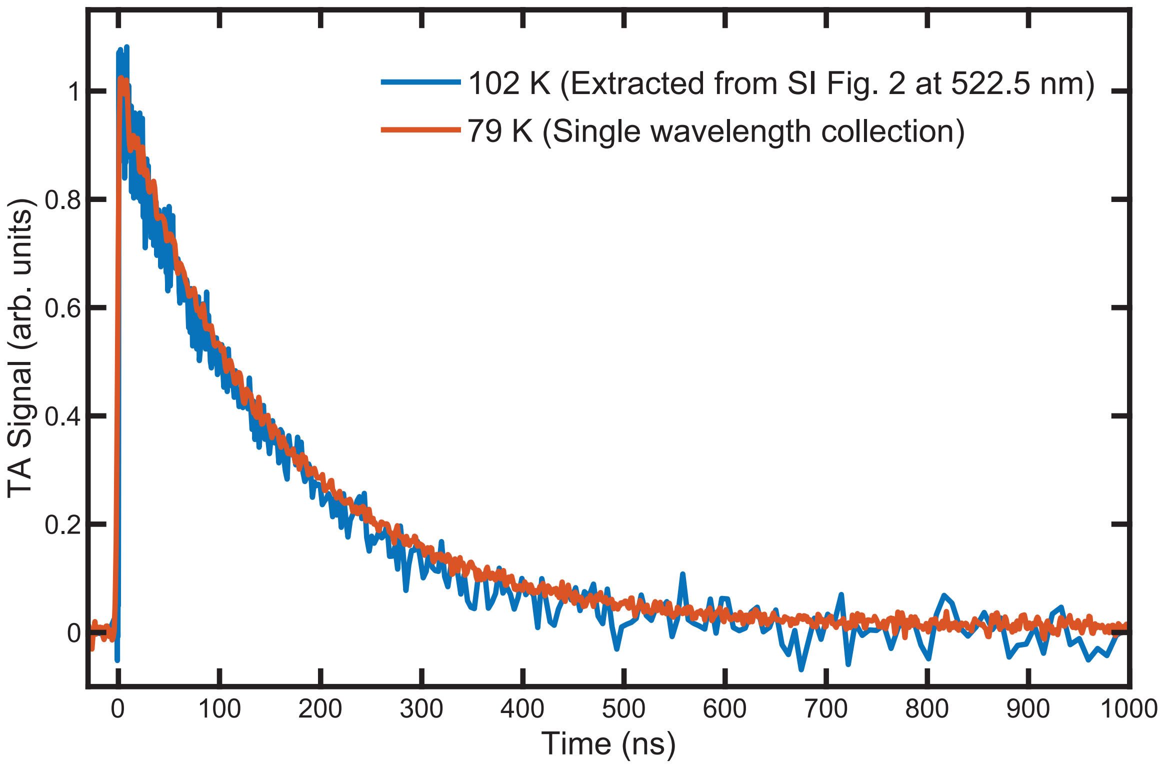

Supplementary Fig. 4: Transient absorption kinetic traces for TIPS-BP1′ in mTHF. The blue trace is a single wavelength kinetic trace selected from the TA data matrix represented spectrally in Supplementary Fig. 2 (522.5 nm probe; 102 K). The orange trace is a single wavelength kinetic trace measured with a monochromator, on a different TA setup, at the same probe wavelength. It is clear from these data that the dynamics at 79 K and 102 K are very similar, which is more easily seen in Supplementary Fig. 3

1.3 trEPR Spectroscopy

Unless otherwise specified, trEPR data were collected at X-band (9.73 GHz) with a Bruker ELEXSYS E 580 in transient mode (CW microwave source) equipped with a dielectric resonator (Bruker EN 4118X-MD4, Q $\approx$ 3500) and a closed cycle helium cryostat (Bruker / Cold Edge Technologies) for low temperature operation. The microwave attenuation was 22 dB, giving an input microwave power of 0.95 mW. Optical excitation (640 nm, 10 Hz repetition rate, 3.5 mJ/pulse, 5 ns FWHM) was provided by an optical parametric oscillator (Opotek Radiant SE 355 LD), coupled to a reflective collimator (Thorlabs RC08SMA-P01), through a 9 meter long optical fiber with 1 mm diameter. The collimator was mounted to the cryostat with a custom mount, such that the collimated beam (diameter $\approx$ 8.5 mm) was directed through the cryostat's optical window at the resonator's optical window. The sample was prepared with a concentration of 74 μM (A(634 nm) = 0.64 in a 2 mm path length cuvette), transferred to a homemade 4 mm outer diameter quartz EPR tube (1 mm wall thickness, Quartz Scientific, Inc.), degassed by several freeze-pump-thaw cycles, and flame-sealed under vacuum with an oxyhydrogen torch. Samples were stored in the dark, and trEPR data were collected the day following sample preparation.

The default Bruker software (Xepr) permits multi-shot averaging of transient signals at each B_0 step, but does not permit repeating the entire field-sweep. We have found that this procedure does not sufficiently average out low-frequency ($\approx$ 10 kHz) oscillatory background signals, possibly because the background oscillations maintain a relatively stable phase relationship with the 10 Hz laser pulses over short times. To mitigate this problem, we used a custom python script (run through the Xepr API) provided to us by Bruker (Ralph Weber), that permits repeating the entire field-sweep. This procedure also facilitates detection of sample degradation during the experiment. For the TIPS-BP1$'$ data presented in the main text, a total of six two-dimensional scans (time versus field) were collected and averaged together. For the TIPS-Pentacene (TIPS-Pc) data presented in Supplementary Fig. 6, four such scans were collected.

For signal processing, we used MATLAB code written in-house. Raw data were first background corrected along both dimensions to correct for field-dependent and time-dependent background signals. At each field point the transient signal before the laser pulse was averaged to give an offset value which was then subtracted from the corresponding transient. The transient signals at off-resonance field values on the high-field and low-field edges of the data were averaged to give a laser-induced time-dependent background signal, which was subtracted from all transients. The data were then rephased to extract the absorptive signal. After this initial processing, the prompt EPR spectrum for TIPS-BP1$'$ (*Experiment*, Fig. 2a) was obtained by averaging the trEPR spectra (Fig. 1a) from 200-400 ns. Apart from the EPR signal growing in, this time range shows minimal spectral evolution (i.e. shape change), and is early enough to largely precede spin relaxation (Supplementary Fig. 5).

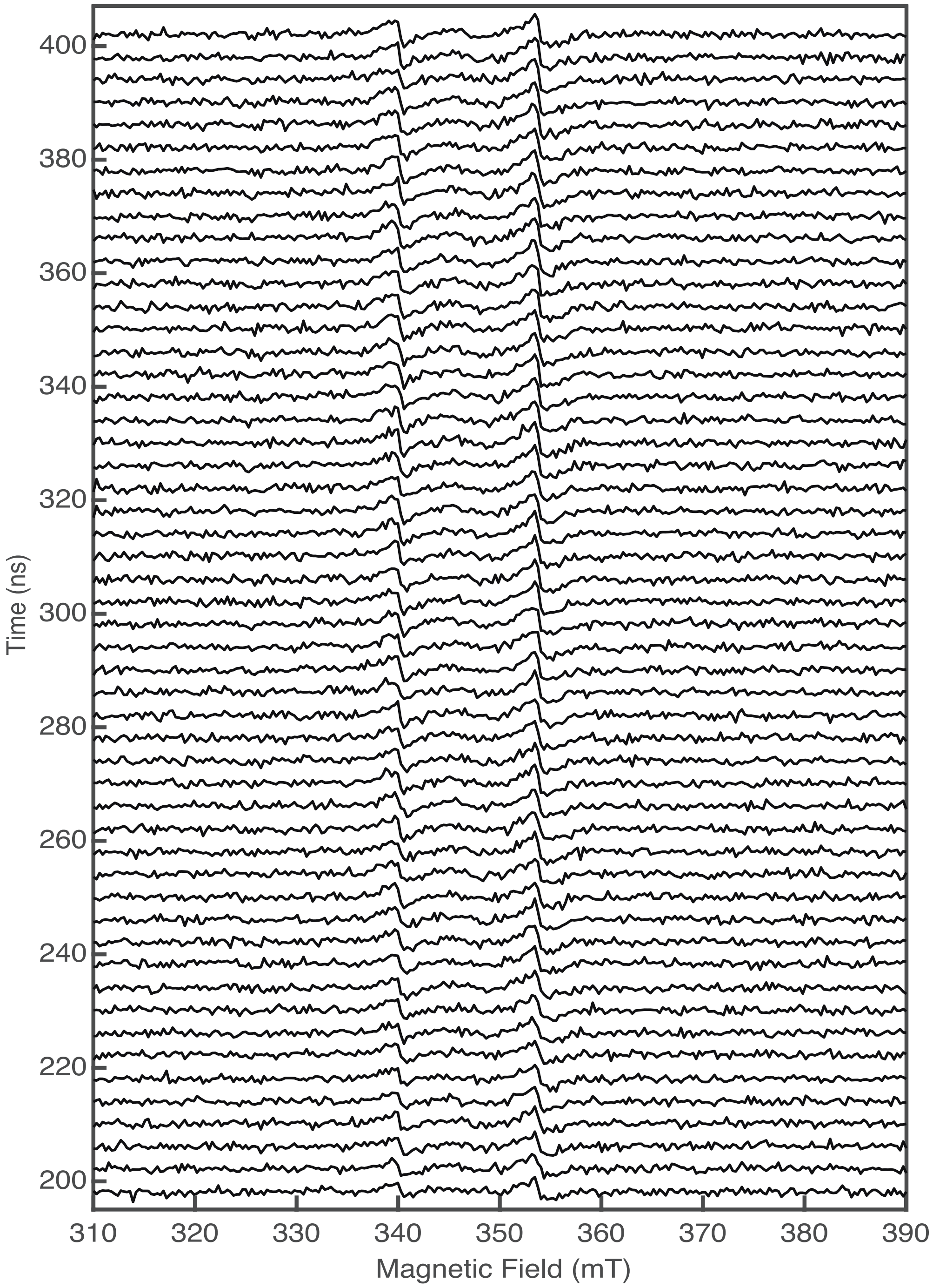

Supplementary Fig. 5: The prompt EPR spectrum for TIPS-BP1′ (Experiment, Fig. 2a, main text) was obtained by averaging the trEPR data (Fig. 1a, main text) from 200-400 ns. Apart from the EPR signal growing in, this time range shows minimal spectral evolution (i.e. shape change), and is early enough to largely precede spin relaxation.

Supplementary Note 2. Supplementary Text

2.1 The intersystem crossing triplet trEPR powder spectrum for TIPS-Pc

For comparison with the dimer trEPR spectrum, we have collected trEPR data for the intersystem crossing triplet of the monomer model TIPS-Pc (Supplementary Fig. 6), using a heavy atom solvent to promote spin conversion. The spectrum is typical of intersystem crossing triplets, and shows an inner peak splitting of 44 mT (1.2 GHz), with a total range of 84 mT (2.4 GHz; from the edge of the low-field emissive shelf to the edge of the high-field absorptive shelf). The spectral feature splittings and range are much larger than those in the TIPS-BP1′ EPR spectrum.

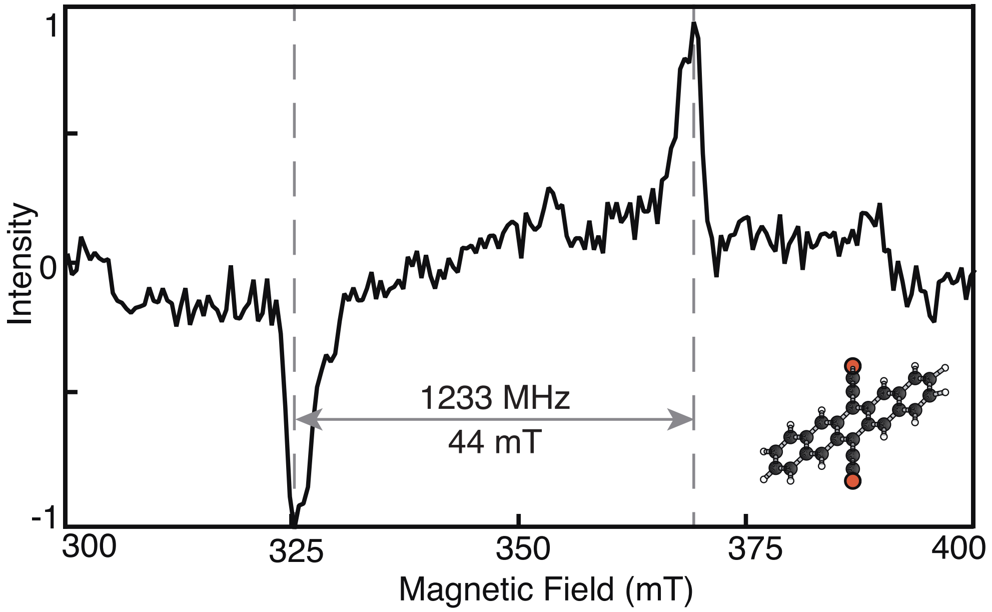

Supplementary Fig. 6: The intersystem crossing triplet EPR spectrum of TIPS-Pc monomers in a heavy atom solvent (4:1 toluene:1-iodobutane, 640 nm excitation, 100 K, $f_{microwave} = 9.74729$ GHz). Because this is a triplet, the splitting between the peaks (dotted grey lines) gives the value of D directly. The measured value of D (1233 MHz), assuming $E = 0$, is similar to the extracted value of D for the TIPS-BP1′ dimer (1322 MHz). The data is an average of scans in the time domain over 101-301 ns, where the signal remained relatively constant. The spectrum appears to be contaminated near 350 mT. We attribute this contamination to the presence of ^{5}TT in aggregated monomers[3], because the signals appear to be split by approximately $|D|/3$.

2.2 Rabi oscillations and the Hankel transform

As described in the manuscript, we have identified that the Rabi oscillations in our trEPR data permit spectral isolation via transform methods, analogous to lock-in detection. We note that these oscillations are best described as a damped Bessel function. This result is derived in Ref. 4 and results from a few simplifying assumptions. First, it describes the system in terms of a Bloch model. Although strictly valid for a two-level system, the description is approximate here. Second, the relationship is valid in the underdamped limit, where $T_2\omega_N \gg 1$, evidently satisfied in our trEPR data. For the data presented in the paper and in Supplementary Fig. 7 ($\omega_N = 4.13$ mrad/ns, $T_2 = 1400$ ns), $\omega_N T_2 = 5.8$. Another data set collected under conditions of higher B_1 (Supplementary Fig. 7, inset) gives a nutation frequency that is 1.7 times larger, such that $\omega_N T_2 = 10$, and yields the same value for T_2. A third assumption is the common one that $T_1 \gg T_2$ so that the decay is not dominated by sublevel population interconversion. A fourth assumption is that the

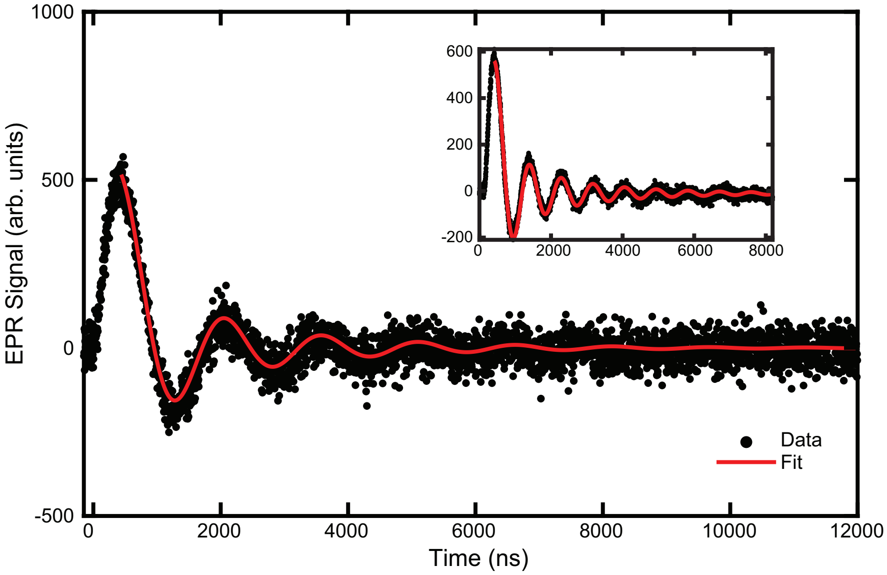

Supplementary Fig. 7: The time-domain trEPR signal decays according to a Bessel Function (see main text). The TIPS-BP1′ trEPR data, averaged from 352.8-353.7 mT, fit to the functional form given by Vollmann and coworkers[4] plus a constant offset: $AJ_0\left(\omega_N\left(t-t_0\right)\right)\times exp(-\left(t-t0\right)/\left(2T_2\right))+C$. Fit parameters: ω_N = 4.13 mrad/ns = 0.657 MHz, T_2 = 1.4 μs. These data are also presented in the main text (Fig. 1a, inset). **Inset:** Another data set taken under conditions of higher microwave power (averaged over the same field range; microwave frequency = 9.7367 GHz), fit to the same functional form. Fit parameters: ω_N = 7.11 mrad/ns = 1.13 MHz, T_2 = 1.5 μs. The coherence times are approximately the same for both data sets.

EPR line's inhomogeneous broadening is significantly larger than the nutation frequency, when converted to comparable units using the relation $\omega_N = (g\beta B_1)/\hbar\sqrt{S(S+1) - M(M+1)}$ for a transition between M and $M+1$. This criterion is easily fulfilled in our sample, and indeed in most disordered samples. For the $M = 0 \leftrightarrow \pm 1$ transitions of a quintet, the nutation frequency seen in our data, $\omega_N = 4.13$ mrad/ns $= 0.664$ MHz, would correspond to a microwave field $B_1 = 0.01$ mT (we assume $g = g_e$, the free electron g-factor).

Because the signal $s(B_0, t)$, decays as a damped Bessel function, it is more natural to decompose $s(t)$ onto J_0 than onto Fourier modes. Indeed, the Fig. 1b inset and Supplementary Fig. 8 show that the Bessel transform (Hankel transform) gives a more sharply defined representation of the spectral data than the Fourier transform does. We extract the spectrum of the oscillating species, $A_N(B_0)$, by recognizing that

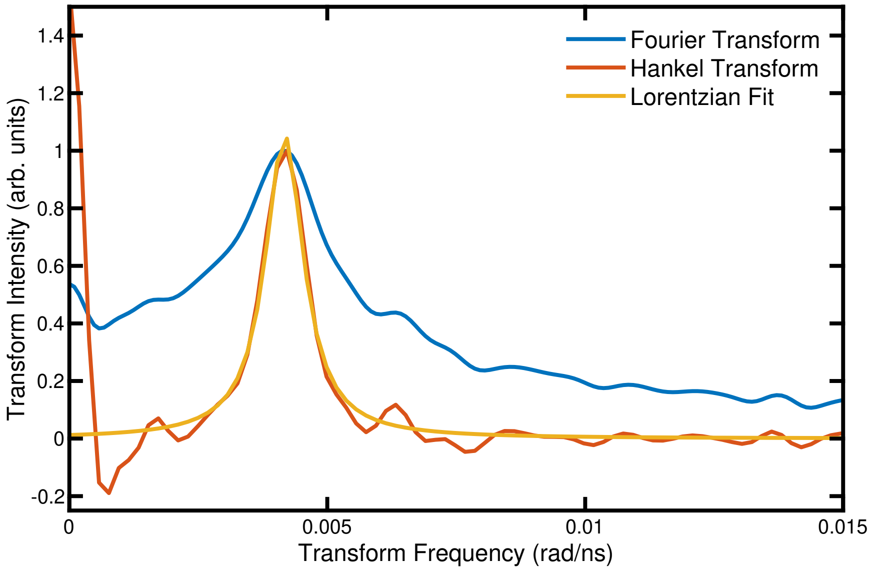

Supplementary Fig. 8: Comparison of the Hankel transform and the FFT amplitude spectrum (square root of the power spectral density) at 353.4 mT. The Hankel transform and the FFT both decompose a time-domain signal into a series of oscillating functions, but the Hankel transform basis is the series of Bessel functions, which are more accurate descriptors of nutating signals than harmonic oscillators are. As a result, the dominant nutation peak is much sharper and more symmetric in the HT spectrum (orange) than in the FFT amplitude spectrum (blue). The HT spectrum fits to a Lorentzian lineshape (yellow) with center frequency $\omega_0 = 4.17$ mrad/ns $= 0.664$ MHz and linewidth $\Gamma = 0.453$ mrad/ns $= 0.0722$ MHz. The center frequency from this fit is used as a normalization factor for the frequency axis in main text Fig. 1b. The fit is also used to determine a weighting function for generating the Hankel spectrum (see below). Both transforms were taken after multiplying the time-domain data with a one-sided Hann window.

$s_N(B_0, t) \propto A_N(B_0) J_0(\omega_N t) e^{(-t/(2T_2))}$, where $s_N(B_0, t)$ is its contribution to the signal. Imitating lock-in detection, we transformed the time-domain signals, $s(B_0, t)$, with the Hankel transform (HT), giving the Bessel frequency-domain signal, $S(B_0, \omega)$, and then performed a weighted average over the nutation band, $A^*_N(B_0) = \sum_i W(\omega_i) \times S(B_0, \omega_i)$. The weighting coefficients, $W(\omega_i)$, are chosen to match the bandwidth of the dominant nutation band centered at ω_N (Supplementary Fig. 8). The weighting function bandwidth is analogous to the low-pass filter bandwidth in a lock-in measurement. The resulting field-swept "Hankel spectrum", $A^*_N(B_0)$, represents the EPR spectrum of the transitions that show nutation within that band.

2.2.1 Hankel Transform

To decompose the trEPR signal, we start by extracting the Hankel spectrum. Because the HT is less familiar than the FFT to most readers, we mention a few important practical differences. First, since zeroth-order

Bessel functions of the first kind start at maxima and are not periodic, the time-domain data before the first extremum must be removed. This introduces a variable time-zero, t_0, into the data. Second, applying the HT to real data gives a purely real result, unlike the Fourier transform which returns real and imaginary parts similar to the cosine and the sine transforms of the data, respectively. In that sense, the HT is similar to the cosine transform, and both are sensitive to t_0. Third, the orthonormality condition for Bessel functions contains a weighting function that is not constant, as it is for plane waves. The Hankel transform of a function f is $H_\omega(f) = \int dt\, t J_0(\omega t) f(t)$, and the Fourier transform of f is $F_\omega(f) = \int dt\, e^{i\omega t} f(t)$. The extra factor of t inside the integrand amplifies both signal and noise at long times for H compared to F. Apodization, or windowing, in the FFT is common practice but it is necessary in the HT. We apply the Hann window to the time-domain data before taking the HT to avoid amplifying noise at long times, which is more prevalent in the HT than it is in the FFT.

To compute the HT spectra (main text Fig. 1b, vertical axis) from the time-domain data (Fig. 1a, main text), we first cropped the data matrix at $t_0 = 401$ ns to eliminate rise dynamics. Next, we windowed the cropped time-domain signal by multiplying it by a one-sided Hann window of 10 μs duration, and then zero-padded the result so that the number of data points is a power of two, 2^{14}. We then applied the HT to the time-domain trEPR signal recorded at every B_0, in MATLAB, using the discrete HT implementation in the ht.m function from ref. 5. A Lorentzian fit to the HT trace at 353.4 mT (Supplementary Fig. 8) found $\omega_N = 4.17$ mrad/ns $= 0.664$ MHz, which we used to rescale the frequency axis in Fig. 1b.

2.2.2 Spectrum Estimation

EPR spectral slices corresponding to specific HT frequencies can be directly selected from the HT data matrix (Supplementary Fig. 9, blue line). Because the nutation appears as a well-resolved band, a weighted average of all the EPR spectral slices contained within the bandwidth gives a less noisy result (Supplementary Fig. 9, orange line). To generate the Hankel spectrum in Fig. 2 and Supplementary Fig. 9, the weighting function we used was the Lorentzian function in Supplementary Fig. 8, further modified by completely excluding data below 2 mrad/ns and above 6 mrad/ns. The Hankel spectrum will not capture components whose oscillatory components differ substantially—outside the bandwidth of the yellow curve in Supplementary Fig. 8—from ω_N (Supplementary Fig. 10). The residual spectrum—the difference between the total prompt spectrum and the properly normalized Hankel spectrum—corresponds to the intensity in the signal from all transitions that do not oscillate at the nutation frequency.

To extract their contributions to the prompt spectrum, We decompose it into its Hankel spectrum, $A_N^*(B_0)$, and its residual, $R(B_0)$: $A_{prompt}(B_0) = cA_N^*(B_0) + R(B_0)$. We determine the constant c by normalizing $c = A_{prompt}(B_0^*)/A_N^*(B_0^*)$, where the point B_0^* is the maximum common to both A_{prompt} and A_N^*. Here we are assuming that when the prompt spectrum is maximal, the intensity is dominated by the Hankel component oscillating at ω_N so that, at this point, the residual signal is negligible. Such normalization is conventional in, for example, optical pump-probe experiments. This gives $B_0^* = 353.4$ mT (see Supplementary Fig. 10). The choice of B_0^* does not change the results very much. Varying the value of c within $\pm$ 10% does not substantially alter the extracted residual spectrum.

For a confirmation of the residual spectrum's shape, we describe another way to measure it. Because the Hankel component oscillates in time as $s_N(B_0, t) \propto J_0(\omega_N t)$, its spectral signatures reach a zero-crossing at 1000 ns. Any persistent signals that do not follow $J_0(\omega_N t)$ are essentially isolated by measuring the spectrum at this time. We first smooth the data along the time-axis with a Savitzky-Golay filter, and then select the 1000 ns spectrum. This is shown in Supplementary Fig. 11 and shows good agreement with the residual spectrum generated by simple subtraction.

The Hankel spectrum isolates the EPR spectrum of the $M = 0 \leftrightarrow \pm 1$ transitions, and clearly explains most of the total EPR spectrum. If only the $M = 0$ sublevel was populated, a common assumption, we would expect the Hankel spectrum to look more like the $M = 0 \rightarrow \pm 1$ spectrum in Fig. 1a (main text). With our model, on the other hand, the observed spectrum results from strongly orientation-dependent sublevel populations, which are computed.

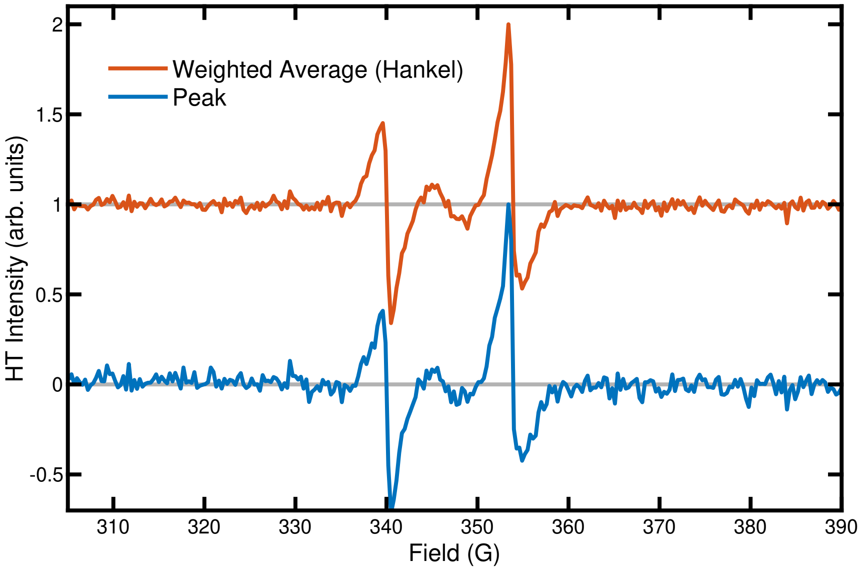

Supplementary Fig. 9: The Hankel spectrum taken from the peak at ω_N (blue), or from a weighted average according to a Lorentzian fit (Supplementary Fig. 8) about ω_N (orange). The Hankel spectrum isolates the EPR spectrum of the quintet $M = 0 \leftrightarrow \pm 1$ transitions oscillating at ω_N. We report the orange curve in Fig. 1b, main text. Compared to the peak spectrum, the weighted average gives a small but significant improvement on the signal-to-noise ratio.

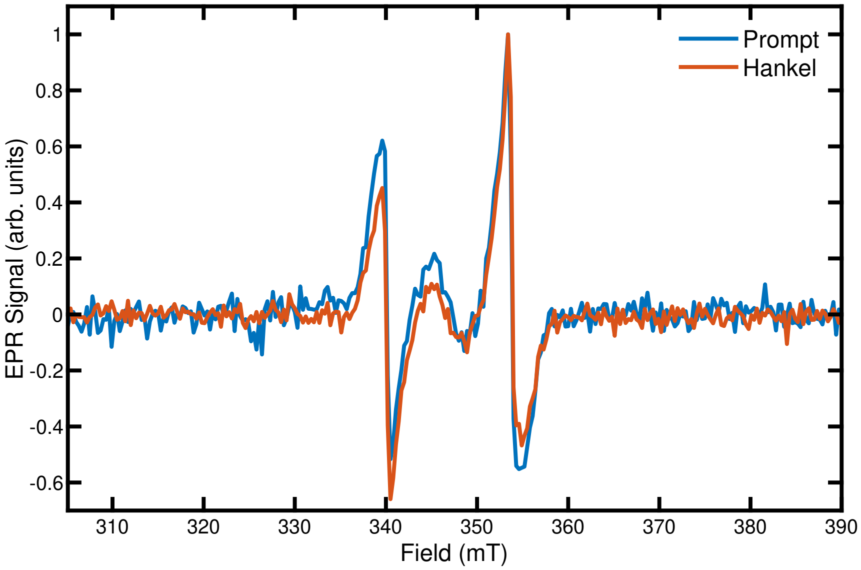

Supplementary Fig. 10: Prompt and Hankel spectra. Both spectra are normalized to have the same magnitude at 353.4 mT. Subtracting the Hankel spectrum from the prompt spectrum gives the residual spectrum reported in Fig. 2c of the main text. The Hankel spectrum ($M = 0 \leftrightarrow \pm 1$ transitions) accounts for most of the intensity in the prompt spectrum.

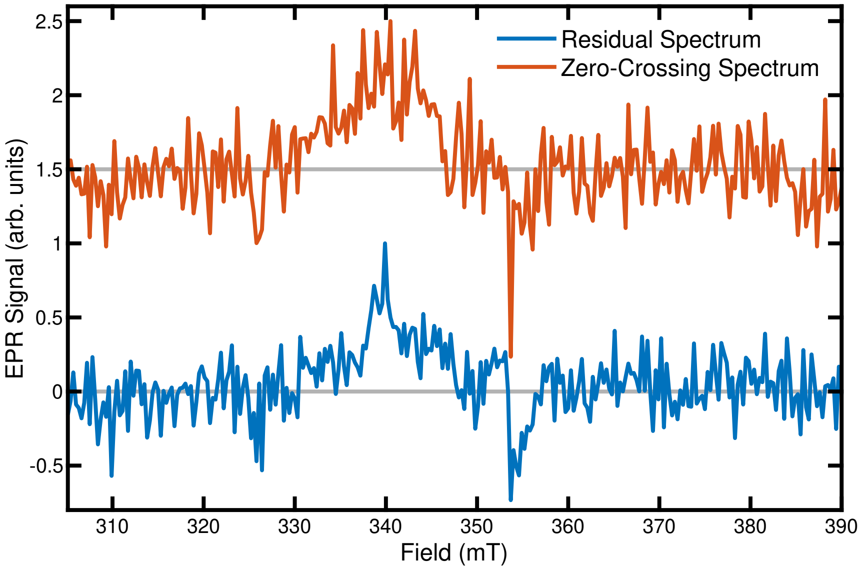

Supplementary Fig. 11: Two different methods for measuring the residual spectrum give very similar results. The residual spectrum (blue) is computed by subtracting the Hankel spectrum from the prompt spectrum normalized at a single B_0 (Supplementary Fig. 10). The zero-crossing spectrum (orange), is the trEPR spectrum at 1000 ns, near the first zero of the Bessel function J_0, after smoothing the data along the time axis with a degree 2, 51-point Savitzky-Golay filter. Near the zero-crossing, the Hankel component contributes little to the observed spectrum. The agreement between these two methods suggests that the uncertainty in determining the residual spectrum is small. We report the residual spectrum (blue) in the main text.

2.3 The Q0 model for the initial population

In literature, it is common to extend Merrifield's rate model for singlet channel fusion [6] to calculate the populations of ^{5}TT sublevels following singlet fission [7,8,9,10]. The first two examples of this [9,10] are equivalent in the limit that spin S is well-defined. Their interpretation says that the populations of the coupled, strong-field triplet pair states $|S, M\rangle$ are proportional to,

$$\begin{aligned} |\langle 00|S,M\rangle|^2 + \frac{1}{2}\,|\langle +-|S,M\rangle + \langle -+|S,M\rangle|^2 \\ = \frac{1}{3}\left|\langle {}^1\mathrm{TT}|S,M\rangle + \sqrt{2}\langle {}^5\mathrm{TT}_0|S,M\rangle\right|^2 \\ + \frac{1}{3}\left|-\sqrt{2}\langle {}^1\mathrm{TT}|S,M\rangle + \langle {}^5\mathrm{TT}_0|S,M\rangle\right|^2, \end{aligned} \tag{S1}$$

noting that

$$\begin{aligned} |00\rangle &= \frac{1}{\sqrt{3}}|{}^1\mathrm{TT}\rangle + \sqrt{\frac{2}{3}}|{}^5\mathrm{TT}_0\rangle \\ \frac{1}{\sqrt{2}}\left(|+-\rangle + |-+\rangle\right) &= -\sqrt{\frac{2}{3}}|{}^1\mathrm{TT}\rangle + \frac{1}{\sqrt{3}}|{}^5\mathrm{TT}_0\rangle, \end{aligned} \tag{S2}$$

where $|00\rangle$, $|+-\rangle$, and $|-+\rangle$ are the uncoupled biexciton Zeeman states $|M_A, M_B\rangle$, and $|S, M\rangle$ and $|^{2S+1}\mathrm{TT}_M\rangle$ are used interchangably to denote the coupled Zeeman states, i.e., $|^1\mathrm{TT}\rangle \equiv |S = 0, M = 0\rangle$. Borrowing notation from ref. 10, we refer to Supplementary Equation (S1) as the 'Q_0' model. Upon inspection of Supplementary Equation (S1), it is clear that only ^{1}TT and $^5\mathrm{TT}_0$ are predicted to have non-zero population, regardless of orientation. Figs. 2a and 3 show that this does not hold for our system. This motivates the need for a theory such as ours for strongly coupled dimers.

2.4 The spin hamiltonian

The model hamiltonian and theory for spin exciton dynamics that we use to model the trEPR spectra for molecular dimers is the *JDE* model [11]. The hamiltonian—and others similar to it—have appeared in several other places in the literature [12,10,13,9,8]. The name of the hamiltonian comes from its parameterization in terms of the isotropic Heisenberg-Dirac exchange coupling between excitons on different chromophores, J, and the anisotropic spin-dipole hamiltonian for excitons on single chromophores, parameterized by D and E.

We extend the previously reported *JDE* model [11] to a disordered sample of non-parallel, yet rigid, dimers to calculate the powder EPR spectra for the quintet triplet pair from TIPS-BP1′. An anisotropic inter-chromophore interaction $\mathbf{X}$ was introduced to the hamiltonian to reproduce the observed spectrum (see following section). We express the hamiltonian in the lab frame,

$$\mathcal{H} = g\mu_B B_0\left(S_{Az} + S_{Bz}\right) + J\mathbf{S}_A\cdot\mathbf{S}_B + \mathbf{S}_A^{\intercal}\cdot\mathbf{X}\cdot\mathbf{S}_B + \mathbf{S}_A^{\intercal}\cdot\mathbf{D}_A\cdot\mathbf{S}_A + \mathbf{S}_B^{\intercal}\cdot\mathbf{D}_B\cdot\mathbf{S}_B, \tag{S3}$$

where $\mathbf{S}_A$ and $\mathbf{S}_B$ are the single-chromophore spin operators (for chromophores A and B), $\mathbf{D}_A$ and $\mathbf{D}_B$ are the zero-field splitting tensors, and $\mathbf{X}$ is the inter-chromophore anisotropic interaction tensor.

We first choose relevant reference frames in which to define the interaction tensors. $\mathbf{D}'_A$ and $\mathbf{D}'_B$ are defined with respect to their principal frames, which are assumed to coincide with chromophore A and chromophore B's molecular frames, respectively. In the (single-chromophore) principal frames,

$$\begin{aligned} \mathbf{D}'_A &= \mathbf{D}'_B = \mathbf{D} \\ &= \begin{pmatrix} -D/3+E & 0 & 0 \\ 0 & -D/3-E & 0 \\ 0 & 0 & 2D/3 \end{pmatrix} \end{aligned} \tag{S4}$$

The inter-chromophore anisotropic exchange interaction $\mathbf{X}$ is defined with respect to the dimer frame—the unit vector sum of the single-chromophore axes (Fig. 3b). We then rotate these interaction tensors from the frames in which they are defined into the lab frame, defined by $\mathbf{B_0} = (0, 0, B_0)$ (Fig. 3c).

Although there are countless ways to do this, we choose to perform the following series of rotations so that the final transformation into the lab frame relates $\mathbf{B}_0$ to dimer axes. This unveils symmetries in the system and allows us to use intuition based on single-triplet EPR spectra when analyzing the ^{5}TT spectra. First, the single-chromophore interactions are rotated into the dimer frame by opposing active (A) rotations about their principal y-axes (short acene axis parallel to ring system). The rotations are a function of the bridging angle β, defined in Fig. 3b, $R_A(0, \pm(180^o - \beta)/2, 0)$. Then, a passive ($P$) rotation re-expresses all anisotropic interactions with respect to the lab frame (Fig. 3c), $R_P(\theta, \phi)$,

$$\begin{aligned} \mathbf{D}_A &= R_P(\theta,\phi) R_A\left(0, \left(180^o - \beta\right)/2, 0\right) \mathbf{D} R_A^{\intercal}\left(0, \left(180^o - \beta\right)/2, 0\right) R_P^{\intercal}(\theta,\phi) \\ \mathbf{D}_B &= R_P(\theta,\phi) R_A\left(0, -\left(180^o - \beta\right)/2, 0\right) \mathbf{D} R_A^{\intercal}(0, -(180^o - \beta)/2, 0) R_P^{\intercal}(\theta,\phi) \\ \mathbf{X} &= R_P(\theta,\phi)\mathbf{X} R_P^{\intercal}(\theta,\phi). \end{aligned} \tag{S5}$$

We refer the interested reader to Mueller for more information on passive and active rotations[14]. Although this is an excellent reference, we note that there is a sign error in Table 1, $d_{pq}^{(k)} = (-1)^{q-p} d_{qp}^{(k)}$, though it is irrelevant to the Cartesian rotations in our work).

2.5 The inter-chromophore anisotropic interaction

Similar to the zero-field splitting interaction $\mathbf{D}$, but for two spin-1 centers, the anisotropic inter-chromophore interaction $\mathbf{X}$ is conventionally derived from the classical dipole-dipole expression. After promoting operators to their quantum mechanical analogs and integrating over spatial wavefunctions, we arrive at the expression for matrix elements of the anisotropic inter-chromophore hamiltonian in the total spin basis[15],

$$\langle S', M' | H_X | S, M \rangle = \langle \mathbf{S}_A^{\intercal} \cdot X \left(\mathbf{1} - 3\hat{r}\hat{r}^{\intercal}\right) \cdot \mathbf{S}_B \rangle \quad = \langle \mathbf{S}_A^{\intercal} \cdot \mathbf{X} \cdot \mathbf{S}_B \rangle. \tag{S6}$$

Here, $\mathbf{S}_A$ and $\mathbf{S}_B$ denote the individual chromophore spin operators that sum to the total spin $\mathbf{S} = \mathbf{S}_A + \mathbf{S}_B$. X describes the magnitude of the interaction which goes as $g^2\mu_B^2/r^3$, where r is the magnitude of the vector $\mathbf{r} = r\hat{r}$ that points from spin center A to B,
$\hat{r} = (\sin\Theta\cos\Phi, \sin\Theta\sin\Phi, \cos\Theta)$, where Θ and Φ are the polar and azimuthal angles that define the orientation of $\hat{r}$ in the chosen coordinate system. We choose to define the anisotropic inter-chromophore interaction tensor in the dimer axis system so that Θ and Φ are analogous to the θ and ϕ shown in Fig. 3c, but they describe $\mathbf{r}$ instead of $\mathbf{B}_0$. In the dimer frame, $(\Theta, \Phi) = (90°, 180°)$, $\hat{r} = (-1, 0, 0)$ and $\mathbf{X}$ is diagonal,

$$\begin{aligned} \mathbf{X} &= X \begin{pmatrix} 1 - 3\sin^2\Theta\cos^2\Phi & -3\sin^2\Theta\sin\Phi\cos\Phi & -3\sin\Theta\cos\Theta\cos\Phi \\ -3\sin^2\Theta\sin\Phi\cos\Phi & 1 - 3\sin^2\Theta\sin^2\Phi & -3\sin\Theta\cos\Theta\sin\Phi \\ -3\sin\Theta\cos\Theta\cos\Phi & -3\sin\Theta\cos\Theta\sin\Phi & 1 - 3\cos^2\Theta \end{pmatrix} \\ &= X \begin{pmatrix} -2 & 0 & 0 \\ 0 & 1 & 0 \\ 0 & 0 & 1 \end{pmatrix}. \end{aligned} \tag{S7}$$

2.5.1 Deriving the *JDE* model with non-zero *X*

In our derivation of the parallel JDE model[11], we showed how the general, bilinear hamiltonian that describes *all* electron-electron interactions ($i, j = 1 - 4$) reduces to,

$$\begin{aligned} \mathcal{H} &= \mathbf{S}_A \cdot \mathbf{O}_{AB} \cdot \mathbf{S}_B + \mathbf{s}_1 \cdot \mathbf{O}_{12} \cdot \mathbf{s}_2 + \mathbf{s}_3 \cdot \mathbf{O}_{34} \cdot \mathbf{s}_4 \\ &= H_{AB} + H_A + H_B, \end{aligned} \tag{S8}$$

where $\mathbf{O}_{ij}$ is a dyadic tensor containing all spin-spin interactions, $\mathbf{S}_A = \mathbf{s}_1 + \mathbf{s}_2$ for chromophore A and $\mathbf{S}_B = \mathbf{s}_3 + \mathbf{s}_4$ for chromophore B. We then showed how each interaction tensor breaks down into a sum of rank-0, rank-1 and rank-2 irreducible parts. To model the TIPS-BP1′ spectrum, we again neglect the large intra-chromophore isotropic interaction (~ 1 eV) and all rank-1 interactions ($\ll |D|$) so that $\mathbf{O}_{12} = \mathbf{O}_{34} = \mathbf{D}$ (Supplementary Equation (S4)). By calculating the spectrum for TIPS-BP1′, we found that it was necessary to retain the rank-2 inter-chromophore anisotropic interaction to replicate the observed lineshape. In this case, $\mathbf{O}_{AB} = J\mathbf{I} + \mathbf{X}$, where $\mathbf{I}$ is the identity matrix.

We urge caution that this interaction only simplifies to the form commonly referenced in singlet fission literature[16] when the vector connecting chromophore pairs is in the yz-plane of the chosen coordinate system. For example, considering the convention outlined above, when $\Phi = 90°$. Furthermore, we stress that the representation of this interaction, as derived in Supplementary Equation (S8), is irreducible—its matrix form is traceless and symmetric. (Note, a typo in the Supporting Information from ref. 9 gives an $\mathbf{X}$ with non-zero trace, and it has propagated through literature[12]).

2.6 Calculating EPR spectra

Spectral fitting and calculations were performed with in-house code written in the Julia programming language (https://github.com/joeleaves/JDE.jl). The spectra in Fig. 2 were calculated with the JDE model—a nonadiabatic transition theory and model hamiltonian (Supplementary Equation (S3)) for the triplet pair from singlet fission dimers. To calculate the resonances and intensities that make up the quintet powder spectrum, we first calculate a set of hamiltonians for specific dimer orientations that are equally distributed on a sphere[17]. The symmetry of the spin interactions for the structurally well-defined TIPS-BP1′ dimer allows us to truncate the sampled range to the area $\phi \in (0°, 90°)$ and $\theta \in (0°, 90°)$ (Figs. 3d and 3e). For each orientation, we find the four field values of B_0 that satisfy $\Delta E(B_0) = g\mu_B B_1$, where B_1 is the magnitude of the applied microwave frequency and $\Delta E(B_0)$ is the energy gap between the magnetic sublevels of the four allowed transitions. These sublevels belong to one of two relevant bases, $\{|S, \alpha\rangle\}$ or $\{|S, M\rangle\}$ (see Supplementary Note 2.6.1).

Specifically, the quintet EPR intensities are computed from a sum over states formula based on the Golden Rule,

$$I(B_0; \beta, D, X) = A \sum_{\theta,\phi} w(\theta, \phi) \sum_{\alpha,\alpha'} |\langle {}^5\mathrm{TT}_\alpha | S_x | {}^5\mathrm{TT}_{\alpha'}\rangle|^2 \, (p_\alpha - p_{\alpha'}) \, \mathcal{G}\left[\mu_B g B_1 - |\epsilon_\alpha - \epsilon_{\alpha'}|\right], \tag{S9}$$

where α either indexes the adiabatic states or is replaced by M when choosing the diabatic basis. The x-component of the total spin angular momentum S_x is proportional to the transition dipole moment matrix element for the transverse oscillating field B_1. The energy of sublevel α, ϵ_α depends on B_0 and on the spectroscopic parameters β, D, X, and on the orientations (ϕ,θ). The populations p_α, $p_{\alpha'}$ come from the nonadiabatic transition theory as described below and in the main text. $\mathcal{G}(x)$ is a numerical approximation to the Dirac delta function, $\mathcal{G}(x) = \frac{1}{\sqrt{2\pi}\sigma} \exp(-x^2/2\sigma^2)$, where in units of magnetic field $\sigma = 0.6$ mT. The spectrum is resolved on a grid of B_0 sampled to match the experimental data. The weight function $w(\theta, \phi)$ comes from discrete samples of the orientations ϕ, θ drawn from the surface of a sphere[17]. A is the amplitude of the spectrum that is constant for all transitions and orientations.

To calculate populations, we apply the JDE model which is founded in perturbation theory[11]. In brief, the hamiltonian (Supplementary Equation (S3)) is first partitioned into a sum of block-diagonal H_0 and off-diagonal parts V. We project out the block-diagonal elements of H by applying the projection operator, $P = \sum_{M,M'} |{}^{2S+1}\mathrm{TT}_{M'}\rangle\langle {}^{2S+1}\mathrm{TT}_M|$. The block off-diagonal elements are then $V = H - H_0$ and they connect states of different S. Off-diagonal elements between same-spin sublevels remain in H_0 and they, like all elements of V, are $\propto |D|$ and $|X|$. In the diabatic treatment, they are inconsequential. In the adiabatic treatment, we diagonalize the same spin blocks so that the off-diagonalize elements in H_0 dress the diagonal. Then, the transformation that takes the same-spin blocks into a diagonal form is also applied to V. Although this mixing is small, it is not zero (Supplementary Fig. 12). The population of a ${}^5\mathrm{TT}_\alpha$ or ${}^5\mathrm{TT}_M$ level is proportional to the coupling matrix element squared, $|\langle {}^1\mathrm{TT}|V|{}^5\mathrm{TT}_\alpha\rangle|^2$ or $|\langle {}^1\mathrm{TT}|V|{}^5\mathrm{TT}_M\rangle|^2$.

The strong interactions, J and B_0, only appear in H_0—they split the energies of the total spin states. V only contains weak perturbations, $\mathbf{X}$ and $\mathbf{D}$, that cause transitions between spin states. We stress that this result is unique for: (1) our chosen frame of reference, where $\mathbf{B}_0$ defines the quantization axis and (2) choosing a total spin S basis. There are then two contrasting cases. If a single-chromophore frame of reference is chosen, the Zeeman interaction is not diagonal in the total spin basis. In this case, V becomes a function of the strong applied field, $B_0 \sim 10$ GHz, and perturbation theory becomes an invalid approach. Likewise if the hamiltonian is expressed with respect to the uncoupled $|S_A, m_A; S_B, m_B\rangle$ states, V is a function of the strong exchange interaction, $|J| \geq 20$ GHz. These distinctions may be insignificant, for example, if $J = 0$ and the populations can be treated as fit parameters, but they are crucial for the limit in which our nonadiabatic transition theory is relevant—where V and the populations are orientation dependent.

2.6.1 Choice of basis for the triplet pair

Rare stochastic fluctuations in the large exchange interaction J rapidly bring different ${}^{2S+1}$TT spin states close in energy so that weak zero-field perturbations, $\mathbf{X}$ and $\mathbf{D}$, can promote population transfer between the singlet and quintet state and, subsequently, the quintet and triplet state [11]. We aim to model the prompt EPR spectrum, where the ^{1}TT population has already evolved into an EPR active state that in turn remains steady over a given period of time (Supplementary Fig. 5). Under the conditions, $|J| \gg |D| \gg |X|$, we refer to the observed spin as being "well-defined," meaning that the states that describe the triplet pair do not have mixed-spin "character."

The blue spectra in Figs. 2b, 2c, and Supplementary Fig. 12 were calculated by evaluating the spin hamiltonian (Supplementary Equation (S3)) in the Zeeman, or diabatic, basis $\{|S, M\rangle\}$, where S and M are the total spin and the total spin projection: $S = 0, 1, 2$ and $M = 0, \pm 1, \pm 2$.

The red spectrum in Fig. 2a and Supplementary Fig. 12 was calculated by evaluating the hamiltonian (Supplementary Equation (S3)) in the eigenbasis of the quintet subspace. These states are linear superpositions of the diabatic states for $S = 2$, $|S = 2, \alpha\rangle = \sum_M \alpha_M |S = 2, M\rangle$. By restricting diagonalization to a specific spin subspace, S remains well-defined in contrast to the commonly used mixed-spin eigenstates of the full hamiltonian.

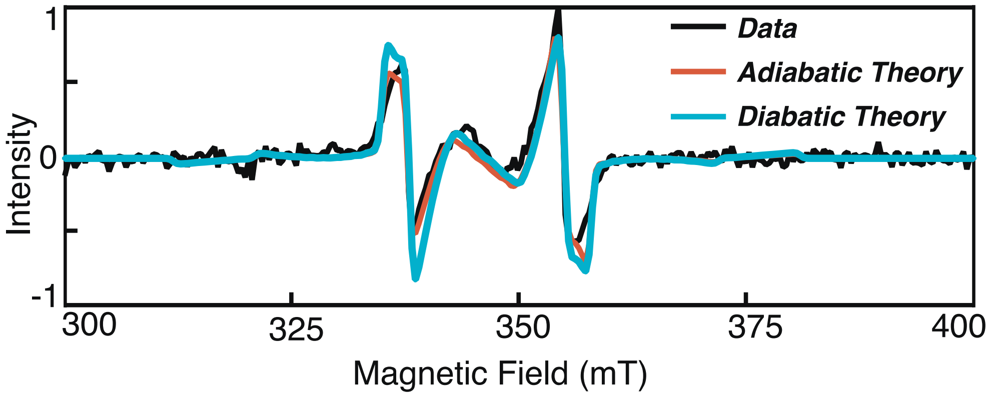

Supplementary Fig. 12: The ^{5}TT spectrum calculated with the JDE model in the diabatic $|S, M\rangle$ basis (blue) is a good fit to the early time trEPR data (black). The calculated spectra and data in Figs. 2b and 2c sum to give the blue and black lines here, respectively. The diabatic spectrum closely resembles the adiabatic spectrum from Fig. 2a (red), though it is symmetric with respect to inversion about center field.

The coefficients $\alpha_M = \langle S, M | S, \alpha_M \rangle$ are functions of the adiabatic interactions, $\mathbf{D}$ and $\mathbf{X}$, and are therefore different for different β, θ, and ϕ (Figs. 3b and 3c). Although the spectrum from the adiabatic states more closely resembles the data, the diabatic spectrum is still a good fit (Supplementary Fig. 12). By expressing the hamiltonian in the diabatic basis, we can assign specific spectral features to transitions between specific states—even in an unoriented sample. This is a significant advantage for quantum computing applications.

2.7 Best-fit parameters by simulated annealing

The best-fit parameters in Supplementary Table 1 provides an approximation to the global minimum of the least squares residual, $\chi^2(\beta, D, X) = 1/N \sum_i^N \left[\left(S_{i,\mathrm{data}} - S_{i,\mathrm{model}} \right) / \sigma_i \right]^2$, where S_{model} is the adiabatic spectrum. The calculation was carried out at the same N field points that appear in the data, S_{data} (Fig. 2a). The error in the intensity at each field point, σ_i, was estimated by jackknifing the average of six consecutively acquired data sets for TIPS-BP1′. In practice, we float the amplitude of S_{model} (Supplementary Equation (S9), $A = 0.8$) and the lateral shift of S_{model} by dressing the g-factor (Supplementary Equation (S9), $g = 2.003067$) to compensate for shielding affects and small fluctuations in the microwave frequency.

The SAMIN algorithm from the Optim.jl Julia package[18] was implemented to minimize the objective function, χ^2. Initial values for the floated parameters were chosen at random from a bounded parameter space. $D \in [1000, 2000]$ MHz, $X \in [0, 200]$ MHz, and $\beta \in [107, 115]$ Degree were floated within the specified parameter spaces to optimize the calculated TIPS-BP1′ spectrum. The chosen range for β was based on the optimized structure for the ^{5}TT state of TIPS-BP1′, 110.9°, which is in good agreement with the best-fit value 111.1° returned by the EPR optimization. The initial value for the "temperature" parameter was chosen so that the entire parameter space is accessible.

The optimization parameters r_T (0.85), N_S (20), and N_T (10) were tuned until the minima for 50 runs of the SAMIN algorithm converged. The objective function tolerance was 10^{-3} and the parameter tolerance 10^{-2}.

By comparing the results of repeated simulated annealing runs, we found significant correlation between D and E when fitting the TIPS-BP1′ spectrum. For a non-zero E, D increases linearly. Further, a non-zero value for E ($E < 0$) increased the error associated with D by an order of magnitude. Because including a non-zero E produced an overfitted model, we set E to zero. This result agrees well with the value of E reported for acenes[19].

Supplementary Table 1: Best fit parameters for calculating TIPS-BP1′ spectra.

Fitted parameter	Mean value	Error (±)
D (MHz)	1322	3
X (MHz)	59	1
β (deg)	111.1	0.2

The error is the standard deviation of best fit parameters for four bootstrapped data sets.

Supplementary References

1. Carey, T. J., Miller, E. G., Gilligan, A. T., Sammakia, T. & Damrauer, N. H. Modular Synthesis of Rigid Polyacene Dimers for Singlet Fission. *Org. Lett.* **20**, 457–460 (2018).

2. Gilligan, A. T., Miller, E. G., Sammakia, T. & Damrauer, N. H. Using Structurally Well-Defined Norbornyl-Bridged Acene Dimers to Map a Mechanistic Landscape for Correlated Triplet Formation in Singlet Fission. *J. Am. Chem. Soc.* **141**, 5961–5971 (2019).

3. Chernick, E. T. *et al.* Pentacene Appended to a TEMPO Stable Free Radical: The Effect of Magnetic Exchange Coupling on Photoexcited Pentacene. *Journal of the American Chemical Society* **137**, 857–863 (2015).

4. Furrer, R. *et al.* Transient ESR nutation signals in excited aromatic triplet states. *Chem. Phys. Lett.* **75**, 332–339 (1980).

5. Leutenegger, M. Hankel transform. https://www.mathworks.com/matlabcentral/ fileexchange/13371-hankel-transform (2021).

6. Johnson, R. C. & Merrifield, R. E. Effects of Magnetic Fields on the Mutual Annihilation of Triplet Excitons in Anthracene Crystals. *Physical Review B* **1**, 896–902 (1970).

7. Jacobberger, R. M., Qiu, Y., Williams, M. L., Krzyaniak, M. D. & Wasielewski, M. R. Using Molecular Design to Enhance the Coherence Time of Quintet Multiexcitons Generated by Singlet Fission in Single Crystals. *J. Am. Chem. Soc.* **144**, 2276–2283 (2022).

8. Chen, M. *et al.* Quintet-triplet mixing determines the fate of the multiexciton state produced by singlet fission in a terrylenediimide dimer at room temperature. *Proc. Natl. Acad. Sci.* **116**, 8178–8183 (2019).

9. Tayebjee, M. J. *et al.* Quintet multiexciton dynamics in singlet fission. *Nat. Phys.* **13**, 182–188 (2017).

10. Weiss, L. R. *et al.* Strongly exchange-coupled triplet pairs in an organic semiconductor. *Nat. Phys.* **13**, 176–181 (2017).

11. Smyser, K. E. & Eaves, J. D. Singlet fission for quantum information and quantum computing: the parallel JDE model. *Sci. Rep.* **10**, 18480 (2020).

12. Lubert-Perquel, D. *et al.* Identifying triplet pathways in dilute pentacene films. *Nat. Commun.* **9**, 4222 (2018).

13. Nagashima, H. *et al.* Singlet-Fission-Born Quintet State: Sublevel Selections and Trapping by Multiexciton Thermodynamics. *J. Phys. Chem. Lett.* **9**, 5855–5861 (2018).

14. Mueller, L. J. Tensors and rotations in NMR. *Concepts in Magn. Reson. Part A* **38A**, 221–235 (2011).

15. Bencini, A. & Gatteschi, D. *Electron Paramagnetic Resonance of Exchange Coupled Systems* (Springer Berlin Heidelberg, 1990).

16. Benk, H. & Sixl, H. Theory of two coupled triplet states. *Mol. Phys.* **42**, 779–801 (1981).

17. Stoll, S. & Schweiger, A. EasySpin, a comprehensive software package for spectral simulation and analysis in EPR. *Journal of Magnetic Resonance* **178**, 42–55 (2006).

18. Mogensen, P. K. & Riseth, A. N. Optim: A mathematical optimization package for julia. *Journal of Open Source Software* **3**, 615 (2018).

19. Swenberg, C. E. & Geacintov, N. E. Exciton interactions in organic solids. In *Organic molecular photophysics*, vol. 1, 489–564 (Wiley, London, 1973).
